# Cognitive Aftereffects of Acute tDCS Coupled with Cognitive Training: An fMRI Study in Healthy Seniors

**DOI:** 10.1155/2021/6664479

**Published:** 2021-04-13

**Authors:** P. Šimko, M. Pupíková, M. Gajdoš, I. Rektorová

**Affiliations:** ^1^Central European Institute of Technology-CEITEC, Masaryk University, Applied Neuroscience Research Group, Brno, Czech Republic; ^2^Faculty of Medicine, Masaryk University, Brno, Czech Republic; ^3^Faculty of Medicine and St. Anne's University Hospital, First Department of Neurology, Brno, Czech Republic

## Abstract

Enhancing cognitive functions through noninvasive brain stimulation is of enormous public interest, particularly for the aging population in whom processes such as working memory are known to decline. In a randomized double-blind crossover study, we investigated the acute behavioral and neural aftereffects of bifrontal and frontoparietal transcranial direct current stimulation (tDCS) combined with visual working memory (VWM) training on 25 highly educated older adults. Resting-state functional connectivity (rs-FC) analysis was performed prior to and after each stimulation session with a focus on the frontoparietal control network (FPCN). The bifrontal montage with anode over the left dorsolateral prefrontal cortex enhanced VWM accuracy as compared to the sham stimulation. With the rs-FC within the FPCN, we observed significant stimulation × time interaction using bifrontal tDCS. We found no cognitive aftereffects of the frontoparietal tDCS compared to sham stimulation. Our study shows that a single bifrontal tDCS combined with cognitive training may enhance VWM performance and rs-FC within the relevant brain network even in highly educated older adults.

## 1. Introduction

The augmentation and restoration of cognitive function among the aging population are an exciting research topic and field of interest. Working memory (WM), especially visual working memory (VWM), deteriorates as a result of aging [1, 2]. WM refers to a temporal buffer for storing and manipulating sensory or abstract information to be for other cognitive processes and is considered to be crucial for executive functions such as planning, reasoning, and decision-making, and also important for everyday functioning [3–5]. Major cognitive brain networks such as the frontoparietal control and dorsal attention networks are important in governing the processes of maintaining, updating, and executing working memory [6–8].

Research aiming to enhance cognition using noninvasive brain stimulation (NIBS) techniques suggests that ongoing cerebral network processing can be tuned and reorganized in a desirable way [9], resulting in favorable behavioral aftereffects via improved inter- and intranetwork communication [10–14]. Transcranial direct current stimulation (tDCS) is a simple, inexpensive, safe, and painless neuromodulation technique capable of enhancing cognition using a weak direct current, typically between a pair of electrodes placed on the scalp, to change the cortical excitability of the underlying brain tissue. It has been proposed that, due to the relatively subtle neuromodulatory effect (the induced electrical field in the brain is <1 V/m, producing minimal cell membrane polarization), the enhancing potential of tDCS may be more pronounced when brain networks are already engaged in cognitive tasks [9].

Brain stimulation in the aging population introduces challenges such as a general lower NIBS response rate, likely associated with an age-related general reduction in neuroplasticity [15], and higher gray matter atrophy that may hinder cognitive gains from stimulation [16, 17]. Despite these challenges, both in healthy aging and in age-related neuropsychiatric conditions such as mild cognitive impairment (MCI) due to neurodegenerative disease, combinatorial approaches such as the combination of NIBS with cognitive or physical training may have significant cognition-enhancing effects [12, 18–27].

The question of identifying the optimal tDCS protocol with an aim of improving VWM in the aging population is an important and still unresolved topic. Several tDCS electrode placements were tested in a single session [12, 23] and multiple session regimens in healthy older adults [11, 13, 24, 25] and in patients with MCI [26, 27]. The most frequently used cortical targets for tDCS in the aging population has been the right dorsolateral prefrontal cortex (DLPFC) [13, 23, 25, 27, 28], the left DLPFC [29, 30], the left inferior frontal cortex [26], and the right parietal (CP5 and P6, respectively, according to 10-10 EEG system) [12, 23] and right parietotemporal (T6 according to the 10-20 EEG system) [11] cortices, known to serve specific roles in WM processing [31, 32]. Whether it is more effective to target the unilateral frontoparietal cortex relative to bifrontal stimulation remains inconclusive. Arciniega et al. [23] used a single session of tDCS with various spatial electrode montages with the aim of enhancing the VWM. In this pilot study, the authors reported that targeting the right frontoparietal cortex (anode over the right DLPFC with cathode over the right posterior parietal cortex) led to more pronounced VWM benefit in older adults (67.72 ± 4.52 years old) compared to bifrontal (anode over the right DLPFC and cathode over the left DLPFC) stimulation. The authors argued that this difference in VWM performance between the montages could be attributed to the restoration of a more “youthful-like” lateralized pattern of brain activity after the unilateral right frontoparietal stimulation. Of note, the authors employed only online memory tasks without further exploration of the tDCS-induced aftereffects. However, the tDCS-induced cognitive aftereffects are of major clinical relevance that may affect daily functioning.

In the current study, we utilized tDCS coupled with the “online” VWM training task and resting-state fMRI to study acute intervention aftereffects on VWM performance in healthy older adult subjects (≥60 years of age) and to identify neural correlates of the changes. Since there is a lack of consensus regarding the electrode placement for inducing the optimal stimulation aftereffects, we used two different electrode montages in a double-blind, randomized, crossover trial. We applied a bifrontal tDCS montage with the anode placed over the left DLPFC, which has been shown to promote frontal compensatory mechanisms in healthy older adult subjects [29] and associated with WM enhancement [29, 33, 34]. The right frontoparietal electrode montage was based on the study by Arciniega et al. [23] and on results from our own neuroimaging fMRI study [35]. In that work, the visual object-matching WM task-induced BOLD signal increases were altered within the right middle frontal gyrus (for amnestic MCI) and in the right posterior parietal lobule (for Parkinson's disease with MCI) as compared to healthy older adults, suggesting deficient top-down modulation of visual processing as a marker of cognitive decline.

Little is currently known about how tDCS affects the large-scale brain networks in older subjects. We decided to investigate the tDCS-induced changes in resting-state functional connectivity (rs-FC) within the frontoparietal control network (FPCN). We selected this network to study since it is engaged in a variety of cognitive control processes, particularly those involving information integration, response selection, spatial attention, and decision making [36]. Its hub nodes were strongly involved in the visual object-matching WM task [35]. Moreover, the electrode placement was within the frontal and parietal hubs of the network, making it more prone to intervention-induced connectivity changes. Based on previous NIBS research, we expected that successful tDCS would lead to intrinsic connectivity enhancement in the network [13, 14].

To our knowledge, this is the first study to examine the behavioral aftereffects of tDCS coupled with “online” VWM training in a healthy older population with the aim of identifying neural correlates of these aftereffects via rs-fMRI analysis.

## 2. Materials and Methods

### 2.1. Subjects and Study Design

Twenty-five healthy older adults (68.84 ± 4.65 years old; 17 women and 8 men) participated in the study. Only participants with no serious neuropsychiatric conditions and with intact cognition were included in the experiments on the basis of a complex neuropsychology examination prior to the study; no participants had ferromagnetic metals in their bodies (due to the presence of MRI data acquisition). All participants had a high school or higher education level of 14.48 ± 2.64 years.

We used a double-blind crossover design. All participants underwent a series of four tDCS stimulations using two distinct electrode montages with corresponding sham stimulation over the same stimulation areas. The study was approved by the local ethics committee. Informed written consent was obtained from participants prior to the study procedures. The trial was preregistered in ClinicalTrials.gov under NCT04134195.

**Figure 1:**
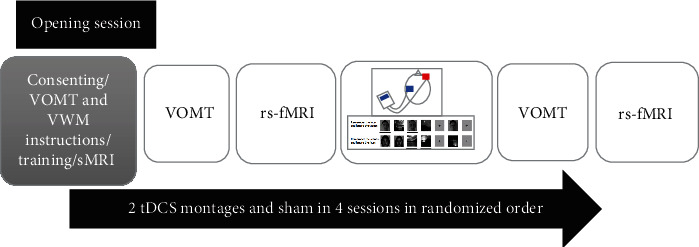
During the opening session, participants underwent sMRI and a neuropsychological examination and they practiced the VOMT and online VWM task. The VOMT and rs-fMRI were performed prior to and after each tDCS.

All participants had functional MRI (fMRI) prior to and immediately after tDCS in each experimental session. The main behavioral outcome, the visual object-matching task (VOMT) was performed before and after the tDCS with a visual working memory task with faces and scenes as an “online” cognitive training task during the tDCS stimulation (online VOMT). All the tasks were practiced by the participants during the baseline (opening) session to prevent high learning effects between the first and the second stimulation session. During the opening session, all participants underwent structural magnetic resonance brain imaging (sMRI) with the aim of using these images for precise targeting of electrodes. For further details, see Figure 1.

### 2.2. Neuropsychological Assessment

Prior to study entry, all participants completed a complex neuropsychological evaluation of cognitive functions in multiple domains, including global cognition, visual-spatial perception, memory (short- and long-term, recognition), attention/psychomotor pace, executive functions, and language functions in order to confirm intact cognition. Daily life activities and depressive symptoms were also assessed. Standardized age and education-based normative scores were calculated for each subject. Participants were recruited for the study only if they scored less than 1.5 SD below the normative scores in all cognitive tests. For further details, see Table 1.

### 2.3. Behavioral Outcomes

**Figure 2:**
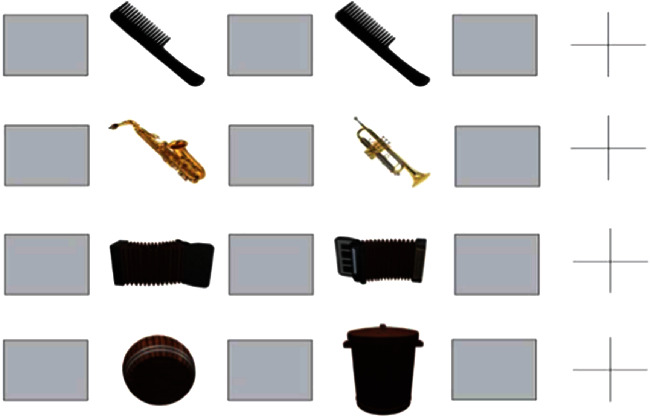
VOMT using conventional and unconventional views of pairs of objects. Line 1: conventional view task condition, correct answer is YES (left button); line 2: conventional view task condition, correct response is NO (right button); line 3: unconventional view task condition, correct response is YES (left button); line 4: unconventional view task condition, correct response is NO (right button).

Visual object-matching task was adapted from Elfmarkova et al. [35]. The main behavioral outcome, VOMT, consisted of 18 pairs of emotionally neutral images of common objects (18 pairs of conventional view images, 18 pairs of unconventional views—spatially rotated, i.e., with one image of the object pair presented in an unconventional rotation, see Figure 2, lines 3 and 4). Every type of event comprised the following sequence: a mask stimulus (1 s), followed by a first object picture (1 s), followed by a mask (1 s), followed by a second object picture (1 s), followed by a mask (1 s), ending with a fixation cross (5 s). Pictures paired in order were presented to the participants. The second image of each pair was either the same (identical) as the first image (conventional, condition 1), different in identity (conventional, condition 2), identical to the first image but spatially rotated (unconventional, condition 3), or different in identity and spatially rotated (unconventional, condition 4). Each of the types of events (conventional conditions 1 and 2 and unconventional conditions 3 and 4) occurred nine times during a protocol. Participants were instructed to respond as quickly as possible by pressing a “YES” (left) button if the second object of the paired images was the same as the first object (regardless of spatial orientation) or by pressing the “NO” (right) button if they were different. After the second image appeared, the participants had to react as quickly as possible. The conditions were randomized. The main outcomes were reaction times (RT; time between the appearance of the stimulus and reaction) and accuracy (the percentage of the correct responses).

**Figure 3:**
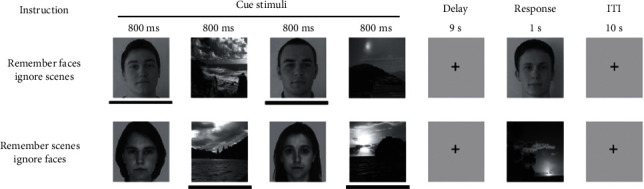
Experimental framework. The two subtasks differ only in the instructions given at the beginning of each run, instructing the participant which, if any, stimuli they should attempt to remember over a 9 s delay, and in the response requirements. In the response period of the two memory tasks, a face or scene stimulus was presented (corresponding to the relevant stimulus class), and participants were required to report with a button press whether the stimulus matched one of the previously presented stimuli.

Visual working memory task with faces and scenes was adapted from Gazzaley et al. [37]. During the tDCS, the participants carried out an “online” VWM cognitive training task consisting of two subtasks in which visual information aspects are kept constant while task demands are manipulated (Figure 3). During each trial, participants watched, in a randomized order, sequences of two faces and two natural scenes. The tasks differed in the instructions, which told the participants how to process the stimuli: (1) remember faces and ignore scenes (“ignore scenes”) or (2) remember scenes and ignore faces (“remember scenes”). In this VWM task, selective attention is required to encode the task-relevant stimuli and the WM is tested after a 9 s delay when the participants are tested on their ability to recognize a sample stimulus as one of the task-relevant cues, yielding a behavioral measurement of visual working memory performance. In this task, together with 48 trials, conditions 1 or 2 were randomized into twelve blocks consisting of eight trials. During each trial, we added 5 s pauses and 30 s pauses between the blocks. The task length was about twenty minutes, copying the tDCS stimulation duration.

### 2.4. Transcranial Direct Current Stimulation

**Figure 4:**
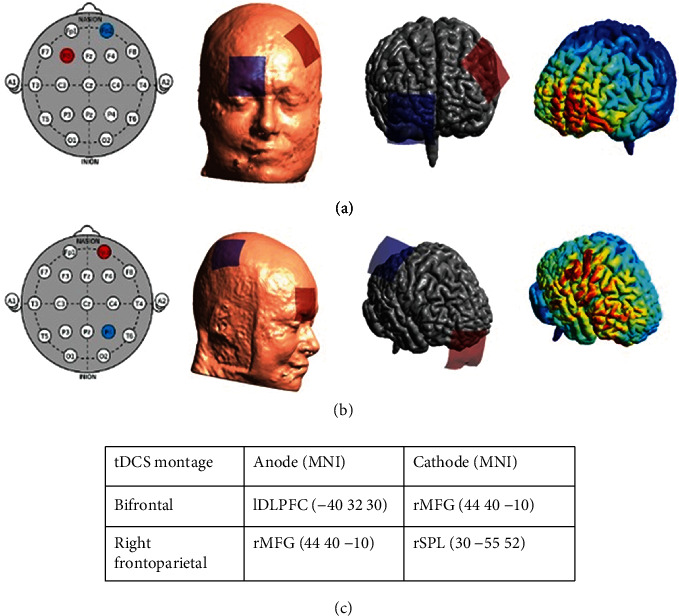
(a, b) Anode and cathode placements for bifrontal and right frontoparietal tDCS montages, respectively, with example current density determined using SimNIBS modeling software [38]; (c) MNI (*x* *y* *z*) electrode-center coordinates for both tDCS montages.

Stimulation using 2 mA (with 30 seconds of ramp-up/down) of direct current was administered in two separate protocols via a battery-driven tDCS stimulator (neuroConn DC stimulator plus from neuroCare Group GmbH, Munich, Germany) with 5 × 5 cm^2^ electrodes attached with conductive gel for 20 minutes—unilateral right and bifrontal stimulation protocol. All participants received four randomized and counterbalanced stimulation sessions (2 distinct montages with corresponding sham stimulation) with at least a 1-day washout period. Using T1-MRI images, the Brainsight™ neuronavigation system was used to precisely locate the middle of the electrodes over the targeted areas. The anode was mounted over the right middle frontal gyrus (MFG) with a cathode attached to the right superior parietal lobule (SPL) for the right-sided unilateral tDCS montage. The anode was placed over the left DLPFC and the cathode over the right MFG in the bifrontal stimulation protocol (for more details on the montages, see Figure 4).

### 2.5. Behavioral Data Analysis

For the behavioral statistical data analysis, we applied a linear mixed model (LMM) with time (baseline vs. poststimulation as time points), stimulation type (real vs. sham), and time × stimulation type interaction as fixed effects and subjects as random effects. The baseline performance was calculated for each subject as an average of the prestimulation performance for each type of tDCS montage (right frontoparietal and bifrontal). Correlations between neuroimaging and behavioral data were performed using the Pearson correlation coefficient. For the correlation analysis, behavioral performance changes were calculated as a postbaseline performance difference. Cohen's *d* for repeated measures was used to estimate the effect size via G-power 3.1 software. The behavioral statistical analysis was performed in IBM SPSS 26.

### 2.6. MRI Data Acquisition and Preprocessing

The MRI data was acquired via 3.0 T Magnetom Siemens Prisma. For the sMRI data, the T1 MPRAGE sequence (TR 1620 ms; TE 2.44 ms; voxel size 1 × 1 × 1 mm; FoV 256 × 256 mm; flip angle 8°; 224 transversal slices) was used; gradient-echo, T2 echo-planar imaging sequence was used for the rs-fMRI data (TR 850 ms; TE 35.2 ms; voxel size 2 × 2 × 2 mm; FoV 208 mm; flip angle 45°; 80 transversal slices; 700 scans; multiband factor 8). During the acquisition of rs-fMRI data, all subjects were instructed to close their eyes and to try not to think about any specific subject while not falling asleep.

We analyzed rs-fMRI data with SPM12 running under MATLAB R2019a. The data preprocessing pipeline included realign and unwarp, spatial normalization, and spatial smoothing (FWHM 5 mm). We controlled data for spatial abnormalities (e.g., dropouts) with the Mask Explorer tool [39] well as for artifacts due to excessive movement using framewise displacement (FD) with the criterion FD < 0.5 mm in less than 20% of scans (5 subjects excluded) and FD < 1.5 mm in any scan (if present, usually no more than 1 scan per session; excluded from analysis). Data were filtered for motion effects (24 motion parameters), for nuisance signals originating in white matter and cerebrospinal fluid, and for high-pass filter (cutoff 1/128 Hz) for the network connectivity analysis.

### 2.7. MRI Data Analysis

**Figure 5:**
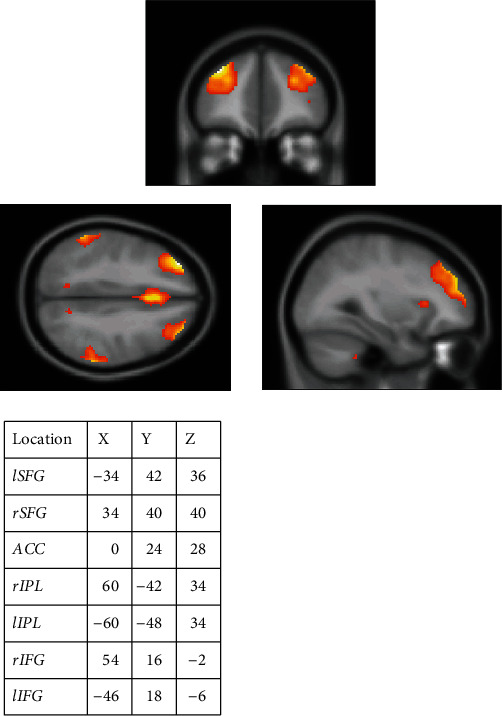
List of selected seeds and their coordinates for the rs-fMRI network analysis. Images decomposed from ICA were used to adjust the seed positions taken from Gao and Lin [36]. Note: lSFG: left superior frontal gyrus; rSFG: right superior frontal gyrus; ACC: anterior cingulum; rIPL: right inferior parietal lobule; lIPL: left inferior parietal lobule; rIFG: right inferior frontal gyrus; lIFG: left inferior frontal gyrus.

After preprocessing, we used independent component analysis (ICA) for preprocessed rs-fMRI data. The data was decomposed into 28 statistically independent components [40, 41] (ICA Spatial Group using ICASSO, infomax algorithm, MDL criterion). The ICA was performed using the GIFT v3.0b toolbox [42] in the MATLAB environment (MathWorks Inc., Natick, MA, US, version R2019a). Manually, we identified spatial components representing the FPCN and chose local maxima from the FPCN regions as regions of interest [36] (see Figure 5).

We extracted fMRI signals from the region of interest spheres (radius 6 mm) around seed positions and used means as representative signals. To analyze connectivity, we used the Pearson correlation between representative signals, converted to *z* values using Fisher *r*-to-*z* transformation. Our focus was on the connectivity between stimulation seeds (seeds placed on the area underneath the electrode center) and the network seeds. Linear-mixed model analysis was applied to evaluate the effect of stimulation, the effect of time, and their interaction on connectivity values. For all the analyses, *p* = 0.05 corrected by the number of tests was used as a level of statistical significance. The analysis was performed in MATLAB (R2019a).

## 3. Results

**Figure 6:**
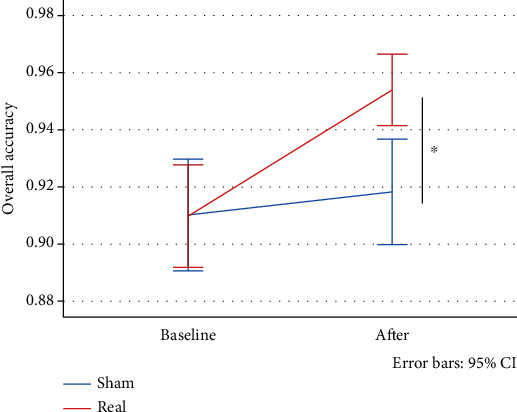
Changes in overall accuracy (in percentage) after bifrontal tDCS montage.

Both tDCS protocols applied in the study were well tolerated and safe. No participants complained about adverse effects such as pain or burning skin sensations during or after stimulation. A few participants reported a mild itching sensation. Regarding the effects of the bifrontal tDCS montage, we found a significant time × stimulation type interaction effect for overall accuracy (*F*(92) = 4.728; *p* = 0.032) favoring the active condition with a medium effect size, *d* = 0.61. The effect of time (*F*(92) = 9.87; *p* ≤ 0.01) and stimulation type (*F*(92) = 4.51; *p* = 0.036) was significant as well, see Table 2 and Figure 6.

For the right frontoparietal tDCS montage, the behavioral results of the VOMT indicated no significant time × stimulation type interaction effect in overall RTs (*F*(96) = 0.234; *p* = 0.63) and overall accuracy (*F*(96) = 0.116; *p* = 0.73).

**Figure 7:**
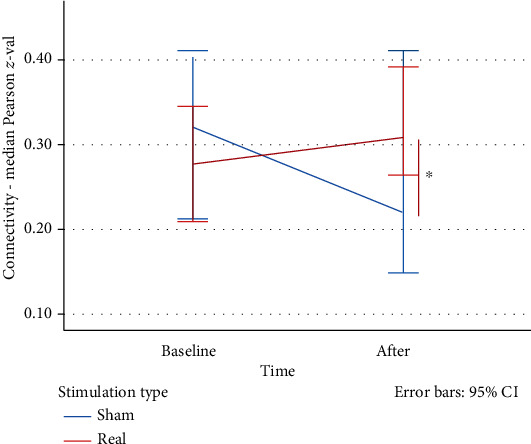
Changes in rs-fMRI connectivity (in percentage) between lDLPFC and IPL using the bifrontal tDCS montage.

Based on the behavioral results, we further explored the neural underpinnings of the bifrontal tDCS utilizing rs-fMRI as described in Materials and Methods. We observed a significant time × stimulation effect on the rs-FC between the anode seed and left inferior parietal lobe (lIPL) (Brodmann area 40: -60 42 34), *F*(73) = 1.592, *p* = 0.024), see Figure 7. The correlation between tDCS-induced behavioral and connectivity changes in the real stimulation condition was not significant (*r*(20) = −0.253, *p* = 0.282).

## 4. Discussion

There are not yet efficient strategies to attenuate age-related cognitive decline. We clearly demonstrated that even a single bifrontal tDCS session with the anode placed over the left DLPFC coupled with online cognitive training led to immediate moderate (effect size of *d* = 0.61) improvement of the performance of a VWM task as compared to cognitive training alone (coupled with sham tDCS). While the general usefulness of tDCS protocols in older adults has been recently challenged [30], our findings are in line with the results of a meta-analysis [43] showing an overall mild to moderate impact of prefrontal tDCS on WM in a healthy aged population. Studies have specifically reported the left DLPFC tDCS efficacy on WM in the same population [29, 33, 34]. Unlike in our study, the tDCS in those studies was not paired with online cognitive training [29, 33] or the study focused only on the online effect of tDCS [28, 34].

A recent study by Nissim et al. [13] also revealed positive effects of a biprefrontal tDCS montage coupled with cognitive training. Of note, the author used inversed electrode polarity, differing from our study (anode over the right DLPFC), and task-induced fMRI (beta values between two regions of interest BOLD time series) instead of rs-fMRI (employed in our study) to identify significant changes in functional connectivity between the seeds engaged in the N-back WM task. Only multiple (10) sessions of tDCS led to both increased letter N-back task accuracy and a functional connectivity increase between the left DLPFC and right inferior parietal cortex after the real as compared to sham stimulation [13] while a single session of tDCS combined with cognitive training did not lead to significant neural or behavioral aftereffects [28]. In line with the results of multiple sessions of tDCS combined with cognitive training [13], our results of a single-session combined intervention also indicate enhanced connectivity between the frontoparietal nodes as a result of real vs. sham tDCS. We focused on the rs-FC changes between the anode seed and the FPCN nodes due to a single session combined intervention, and we observed left-sided functional connectivity enhancement, while Nissim et al. [13] reported interhemispheric connectivity changes as a result of multiple sessions of combined interventions using a different cognitive task, different montage, and different fMRI approach. In other words, the studies by Nissim et al. [13, 28] cannot be directly compared with our results. While both left- and right-sided frontoparietal regions (as assessed by rs-fMRI and graph theory metrics) are related to VWM task performance [44], we believe that the laterality of rs-FC enhancement observed in our study might be tDCS protocol-specific and caused by the left-sided anode placement.

Although we did not observe a correlation between the tDCS-induced behavioral and neural changes, it has been established that the strengthened communication between the frontal and parietal regions represents a likely mechanism of WM processes where the lDLPFC is engaged in top-down attentional control [8] and parietal regions serve as a buffer for WM content [7, 45, 46]. In the context of aging, the major cognitive large-scale brain networks show decreased intranetwork connections, which makes them less distinct [47]. The tDCS-induced increases of the intrinsic FPCN connectivity may reverse decreased local efficiency of the FPCN with age.

Interestingly, no effects were detected after the right-sided frontoparietal tDCS with concomitant cognitive training. This finding is rather incongruent with the proposed hypothesis of the double dissociation of hemispheric specialization for specific WM content [48, 49]; i.e., the right DLPFC tDCS selectively improves visual WM and lDLPFC stimulation enhances the verbal WM. The evidence for the double dissociation comes mainly from experiments conducted on young adults [48, 49]. The role of the lateral prefrontal cortices in WM processing might differ in older adults due to age-related decreased specialization of the brain networks and their nodes [47, 50]. While some studies in older adults support this presumption about the hemispheric specific WM processing [29, 33, 34], other works (including the current study) indicate a more domain-general and modality-unspecific role of the prefrontal and frontoparietal tDCS [13, 23]. The negative result of the right frontoparietal stimulation in our study does not correspond to the results of, e.g., Arciniega et al. [23] and might have been caused by the recruitment of a different group of healthy older adults. We included cognitively well-examined and high-performing volunteers (VOMT close to ceiling, ~90 percent accuracy), while Arciniega et al. [23] showed that only older adults with low WM capacity benefited from the right-sided stimulation. Therefore, it is plausible that only subjects with more pronounced deficits and/or brain pathology might benefit from tDCS with this electrode montage. Further studies should shed light on this assumption.

Taken together, our study further supports the notion that specific tDCS montages may have optimal aftereffects in distinct groups of subjects [23, 51–54]. We clearly demonstrated that even high-performing older volunteers may benefit from bifrontal tDCS with concomitant cognitive training as compared to cognitive training alone (coupled with sham tDCS) and this combined intervention protocol may lead to improved VWM task accuracy and underlying enhancement of the FPCN rs-FC. We are fully aware of some limitations of this study. Our study sample consisted of only older adults with higher education; this is known to mediate WM improvement [51], and thus, our findings may not be transferable to the general population of older adults.

## 5. Conclusion

This randomized controlled study demonstrated that even a single bifrontal session of real tDCS, with the anode placed over the left DLPFC, combined with cognitive training as compared to just cognitive training with sham stimulation, may improve the VWM performance in high-performing healthy older individuals and enhance rs-FC between the left-sided frontoparietal seeds of the FPCN. Future studies should explore whether improvement in an experimental cognitive task may transfer into other tasks and cognitive domains and improve daily functioning. Using multiple sessions of tDCS with simultaneous cognitive training may induce sustained behavioral aftereffects, and dose-controlled tDCS using precise individualized electrical field modeling could be another step forward in the design of future studies.

## Figures and Tables

**Table 1:** Neuropsychological characteristics of the study group, *N* = 25.

Cognitive domain	Test	Mean	SD	Min	Max
Visual perception	JLO	24.36	3.57	17	30
	ROCF-C	33.58	2.05	29	36

Memory	ROCF-I	17.72	4.95	9	27
	ROCF-D	17.14	5.31	5	26
	ROCF-R	20.13	1.81	17	23
	WL-I	30.2	3.91	21	24
	WL-D	5.5	2.3	1	9
	WL-R	22.54	1.35	8	22

Attention, psychomotor speed	TMT-A	39.41	9.64	24	70
	ST-W	81.37	10.79	56	101
	ST-C	68.41	10.41	51	88

Executive functions	ST-CW	37.33	8.33	24	58
	ST-I	2.09	7.16	-16.7	17
	TMT-B	88.04	22.02	52	130
	VFT-S	24.92	6.39	15	38
	VFT-L	41.88	9.47	27	63
	FPT	31.18	9.24	12	51

Language	TT	35.27	0.66	34	36
	BNT-30	27.22	2.02	23	30

Depression	BDI II	9.36	5.5	3	27

Daily functioning	FAQ	0.08	0.39	0	2

JLO: Judgment of Line Orientation; ROCF-C, I, D, R: Rey-Osterrieth Complex Figure Test: copy, immediate, delayed recall, and recognition; WL-I, D, R: Wechsler Memory Scale III: Word List immediate, delayed recall, and recognition; TMT-A: Trail-Making Test part A; ST-W, C, CW, INT: Stroop Color and Word Test, word, color, color-word, and interference score; TMT-B: Trail-Making Test part B; VFT-S, L: Verbal Fluency Test, semantic, lexical; FPT: five-point test; TT: Token Test; BNT-30: Boston naming test; BDI II: Beck's depression inventory.

**Table 2:** Behavioral results in the main behavioral outcome VOMT.

Measure	tDCS setup		Baseline (mean ± SD)	After (mean ± SD)	Factor time *p* value	Factor stimulation type *p* value	Factor interaction *p* value
Accuracy (decimal percent)	Right frontoparietal	Active	0.89 ± 0.04	0.92 ± 0.04	≤0.01^∗^	0.73	0.73
		Sham		0.92 ± 0.04			
	Bifrontal	Active	0.90 ± 0.04	0.95 ± 0.03	0.02^∗^	0.03^∗^	0.03^∗^
		Sham		0.91 ± 0.04			
RT (ms)	Right frontoparietal	Active	1.09 ± 0.26	1.06 ± 0.25	0.99	0.63	0.63
		Sham		1.11 ± 0.39			
	Bifrontal	Active	1.05 ± 0.26	1.06 ± 0.28	0.87	0.98	0.76
		Sham		1.04 ± 0.23			

## Data Availability

The datasets used for supporting the findings in this study are available from the corresponding author on reasonable request.
